# Proteostasis in striatal cells and selective neurodegeneration in Huntington’s disease

**DOI:** 10.3389/fncel.2014.00218

**Published:** 2014-08-07

**Authors:** Julia Margulis, Steven Finkbeiner

**Affiliations:** ^1^Gladstone Institute of Neurological Disease, J. David Gladstone InstitutesSan Francisco, CA, USA; ^2^Department of Neurology, University of California at San FranciscoSan Francisco, CA, USA; ^3^Department of Physiology, University of California at San FranciscoSan Francisco, CA, USA; ^4^Taube/Koret Center for Huntington’s Disease ResearchSan Francisco, CA, USA

**Keywords:** striatum, proteostasis, autophagy, proteasome, Huntington’s disease

## Abstract

Selective neuronal loss is a hallmark of neurodegenerative diseases, including Huntington’s disease (HD). Although mutant huntingtin, the protein responsible for HD, is expressed ubiquitously, a subpopulation of neurons in the striatum is the first to succumb. In this review, we examine evidence that protein quality control pathways, including the ubiquitin proteasome system, autophagy, and chaperones, are significantly altered in striatal neurons. These alterations may increase the susceptibility of striatal neurons to mutant huntingtin-mediated toxicity. This novel view of HD pathogenesis has profound therapeutic implications: protein homeostasis pathways in the striatum may be valuable targets for treating HD and other misfolded protein disorders.

## HUNTINGTON’S DISEASE

Huntington’s disease (HD) is an autosomal dominant neurodegenerative disorder caused by a mutation in the gene encoding the huntingtin (Htt) protein. The mutation is an expansion of CAG repeats that encodes a homomeric polyglutamine stretch in the first exon of Htt. Alleles with 35 repeats put an individual at risk for HD; 40 or more invariably lead to disease. Although mutant Htt (mHtt) is expressed ubiquitously, the key pathological hallmark of HD is the selective loss of striatal medium spiny neurons (MSNs) that express enkephalin and γ-aminobutyric acid (GABA; Graveland et al., 1985). As HD progresses, degeneration also occurs in the cortex and later the globus pallidus and thalamus (Vonsattel et al., 1985; Vonsattel and DiFiglia, 1998).

Huntington’s disease arises from the abnormal accumulation of mHtt. In HD mouse models and human patients, the appearance of visible mHtt aggregates called inclusion bodies (IBs) correlates with the onset of behavioral deficits (Davies et al., 1997). IB formation is restricted anatomically despite ubiquitous expression of mHtt. Many mechanisms attempt to explain selective striatal degeneration – including differential Htt expression, mitochondrial dysfunction, and neurotrophic factor expression – but none accounts for the regional selectivity of IBs.

## IB FORMATION IS AN INDICATOR OF CELLULAR PROTEOSTASIS

Inclusion body formation indicates a mismatch between the production and clearance of aggregation-prone protein. The mechanism of IB formation in HD is unclear, but the length of the polyQ repeat region correlates with the number of IBs in diseased brains (Vonsattel et al., 1985; Becher et al., 1998). While the role IBs play in cellular toxicity has been controversial, evidence suggests that IB formation can be dissociated from neurodegeneration (Klement et al., 1998; Saudou et al., 1998; Kim et al., 1999) and is a coping response to mHtt rather than a direct source of toxicity (Arrasate et al., 2004). In addition to aggregated mHtt, IBs contain ubiquitin, molecular chaperones, and proteasome subunits, suggesting that cells have insufficient capacity to clear misfolded mHtt (Sieradzan et al., 1999; Stenoien et al., 1999; Waelter et al., 2001; Mitra and Finkbeiner, 2008). Further evidence suggests that cells can degrade IBs even after they form: Yamamoto et al. (2000) generated an inducible HD mouse model in which they terminated mHtt production after IBs and behavioral deficits arose. Turning off mHtt production caused IBs to disappear and reversed the behavioral deficits (Yamamoto et al., 2000). Although this study demonstrated that IB formation is reversible, it did not address whether IBs are cleared all at once or dissolved gradually. Later work showed that IBs in mHtt-expressing neurons disappear abruptly (Arrasate et al., 2004; Miller et al., 2010), suggesting that neurons can spontaneously and rapidly metabolize IBs. Autophagy and the ubiquitin-proteasome system (UPS) have been implicated in this metabolism. Thus, the balance between the formation and clearance of IBs can provide insight into the efficiency of proteostasis pathways.

## IB FORMATION IS CELL-SELECTIVE

In HD brains, IBs localize within the nucleus and, more commonly, the neuropil of striatal and cerebral cortical neurons. Other subcortical structures, such as the globus pallidus and the thalamus, exhibit fewer IBs (DiFiglia et al., 1997; Maat-Schieman et al., 1999; Sieradzan et al., 1999). Within the human striatum, IBs are only present in 1–4% of neurons, but are more prevalent in the cerebral cortex, which exhibits less cell death in HD (Gutekunst et al., 1999; Sieradzan et al., 1999). Furthermore, few IBs form in the most vulnerable striatal neurons; only 4% of MSNs exhibit IBs, while 50% of NADPH-positive striatal neurons do (Kuemmerle et al., 1999). Greater neuronal death in the striatum does not explain this differential distribution, as both low- and high-grade cases exhibit similar numbers of striatal IBs (Gutekunst et al., 1999).

Differential mHtt expression within the cortex and striatum may account for different IB levels in these regions. Indeed, in a neuronal culture system, the rate of IB formation was tightly correlated with mHtt levels (Arrasate et al., 2004; Miller et al., 2010). In immunolabeling studies, Htt levels were relatively low in striatal neurons but were uniformly high in cortical pyramidal neurons (Ferrante et al., 1997; Fusco et al., 1999; Sieradzan and Mann, 2001; Gourfinkel-An et al., 2004). However, even when striatal and cortical neurons expressed mHtt equally, cortical neurons formed IBs more readily (Tagawa et al., 2004; Arrasate and Finkbeiner, 2012). Thus, intrinsic differences in how cell types handle misfolded proteins contribute to differences in IB formation.

## PROTEOSTASIS IN THE STRIATUM

Neurons are postmitotic cells that require consistently functional proteostasis pathways. While dividing cells can simply dilute misfolded or aggregated proteins through division and growth, neurons rely on intracellular protein quality control pathways, such as degradation, to maintain protein quality (Eden et al., 2011). In addition, as neurons survive throughout an organism’s lifetime, their proteostasis mechanisms must withstand stressors over time. Misfolded proteins, such as mHtt, stress the proteostasis system, which can dysregulate protein quality control mechanisms and lead to cell death. Striatal MSNs are particularly vulnerable to degeneration and cell death even though mHtt is expressed ubiquitously. Here, we review evidence that striatal MSNs have global changes in proteostasis that render them unable to manage protein misfolding.

### UBIQUITIN PROTEASOME SYSTEM

The UPS degrades misfolded and mutated intracellular proteins. Proteins targeted for degradation are ubiquitinated (i.e., tagged with a polyubiquitin chain; Pickart and Fushman, 2004) and delivered to the proteasome where they are unfolded and hydrolyzed (Goldberg, 2003; Pickart and Cohen, 2004). Originally, mHtt IBs were thought to clog the proteasome (Bence et al., 2001). Later work showed that proteasome function was inhibited prior to IB formation and that IB formation actually improved UPS flux (Bennett et al., 2005; Mitra et al., 2009). These findings suggested that diffuse mHtt – mHtt protein outside of a visible IB – impairs proteasome function. IBs may sequester this diffuse population of protein. Subsequently, Hipp et al. (2012) showed that mHtt does not directly block the proteasome. Instead, they found that misfolded mHtt overwhelmed the chaperone system, leading to misfolding of metastable proteins and increased substrate load which in turn overwhelmed the UPS (Hipp et al., 2012).

**FIGURE 1:**
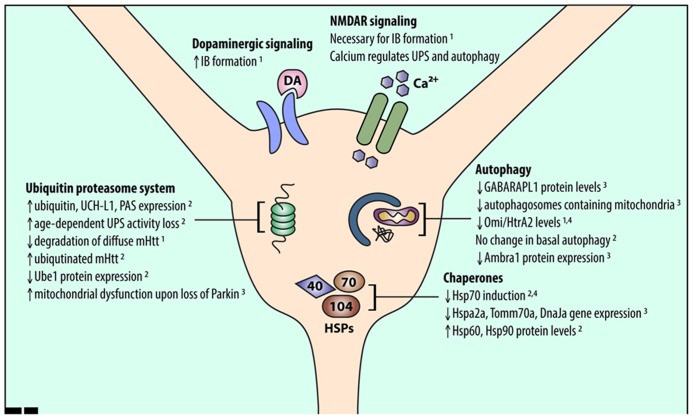
**An overview of alterations in striatal proteostasis pathways.** Protein quality control in neurons is accomplished through three major pathways: the UPS, chaperones and the heat shock response, and autophagy. Recent work indicates that striatal neurons may express and induce the proteins involved in these pathways differently than other cell types. Listed findings were performed in HD and wild-type model systems as follows: (1) primary striatal neuron, (2) HD mouse striatum, (3) wild-type mouse striatum, (4) human HD striatum.

Mutant Htt has differential effects on UPS function in the striatum compared to other brain regions. Levels of a single ubiquitin-activating enzyme, Ube1, are lower in the striatum and cortex than in the cerebellum in CAG140Q knock-in mice (Wade et al., 2014). Conversely, a number of other UPS-associated proteins are upregulated in the striatum and downregulated in the cortex of R6/2 HD model mice (Liu et al., 2007). This upregulation suggests that striatal neurons have an increased need for UPS function, which may make the striatum more susceptible to UPS stressors. Indeed, age-dependent reduction in proteasomal function was shown to be exacerbated in the striatum (Zhou et al., 2003). In addition, global knockout of Parkin, an E3 ubiquitin ligase, resulted in mitochondrial respiration defects and increased oxidative stress in the striatum (Damiano et al., 2014; **Figure 1**).

Recent work also indicates that UPS activity may be lower in the striatum than in the cortex. Tsvetkov et al. (2013) demonstrated that diffuse mHtt is degraded more rapidly in cortical than in striatal neurons. This difference in degradation rate may be due to the UPS, as diffuse mHtt is ubiquitinated (Jana et al., 2001; Waelter et al., 2001; Steffan et al., 2004) and ubiquitinated mHtt accumulates upon proteasomal inhibition in many HD models (Wyttenbach et al., 2000; Jana et al., 2001; Waelter et al., 2001; Lunkes et al., 2002; Zhou et al., 2003). In addition, incubation of mHtt with mouse striatal lysates (compared to cortical or cerebellar lysates) resulted in more ubiquitinated mHtt, pointing to reduced clearance of ubiquitinated mHtt (Wade et al., 2014). Thus, diffuse mHtt may be degraded differently in striatal neurons due to basal differences in striatal UPS function.

### PROTEIN CHAPERONE NETWORK

The protein chaperone network, which includes the heat shock proteins (HSPs), controls cellular protein folding. Since HSPs prevent misfolded proteins from aggregating, target proteins for degradation, and refold misfolded proteins (Sõti et al., 2005; Muchowski and Wacker, 2005; Westerheide and Morimoto, 2005), they may protect against neurodegenerative disease.

Recently, gene expression data from the Allen Brain Institute revealed many chaperone genes that are expressed at different levels in the striatum and cortex, including Hspa2, DnaJa, various Hsp90 co-chaperones, and Tomm70a (Tebbenkamp and Borchelt, 2010). Many of these genes were downregulated in striatum compared to cortex, suggesting reduced capacity for proteostasis stress in striatum. In addition, mHtt expression upregulated Hsp70 in cerebellar neurons, which are largely spared in HD, but not in striatal neurons. Therefore, vulnerable cell populations likely cannot sufficiently upregulate their chaperone system to manage misfolded mHtt (Tagawa et al., 2007). Moreover, in HD mouse models, insufficient activation of HSPs and the heat shock response (HSR) in the striatum was associated with altered chromatin architecture, which reduced access to HSP promoters (Labbadia et al., 2011). The HSR may also be inhibited by proteins that form β-sheets (Olzscha et al., 2011), as mHtt likely does (Thakur and Wetzel, 2002; Poirier et al., 2005). Thus, mHtt misfolding in the striatum may encourage β-sheet-containing mHtt aggregates to form, which inhibit HSPs and further prevent the cell from eliminating mHtt.

Conversely, other work identified HSPs that were upregulated in the striatum and downregulated in the cortex of R6/2 mice (Liu et al., 2007; **Figure 1**). Thus, understanding changes in chaperone protein levels rather than gene expression may help unravel their role in striatal-selective degeneration. Altered gene expression may also not be the only way protein levels are regulated in the brain. Recent studies show that mRNA expression of ribosomal proteins varies across brain regions (Kondrashov et al., 2011; Jackson, 2014), which may explain why striatal chaperone gene and protein expression are not correlated. Further studies are needed to fully unravel HSP network function in striatal neurons.

### AUTOPHAGY

Macroautophagy (hereafter referred to as autophagy) sequesters long-lived proteins, organelles, or parasites within double-membrane autophagosomes (Rubinsztein et al., 2007), which fuse with lysosomes to degrade the sequestered contents. In many cellular and *in vivo* HD models, upregulating autophagy reduces IBs (Qin et al., 2003; Ravikumar et al., 2004; Shibata et al., 2006; Tsvetkov et al., 2010). Thus, autophagy likely regulates IB formation and clearance.

Autophagy-related protein expression varies across brain regions. Le Grand et al. (2013) showed that GABARAPL1, an Atg8 subfamily protein, is highly expressed in the cortex compared to striatum. In another study, wild-type mouse cortex exhibited more mitochondria-containing autophagosomes than did wild-type striatum (Diedrich et al., 2011). Finally, levels of Ambra1, a member of the autophagy core complex, were increased in mouse striatal interneurons compared to MSNs (Sepe et al., 2014). These data indicate that basal levels of autophagy may be lower in susceptible striatal neurons.

Misfolded and aggregated mHtt may impair autophagy induction in striatal neurons. For example, mHtt expression reduces the expression of Omi/HtrA2 in cultured striatal neurons and in human HD striatum (Inagaki et al., 2008). Omi/HtrA2, a mitochondrial chaperone and protease (Clausen et al., 2002), regulates autophagy and mitophagy (Li et al., 2010; Cilenti et al., 2014). Thus, reduced expression of autophagy-related proteins and reduced induction of autophagy may make striatal neurons more vulnerable to mHtt (**Figure 1**). Interestingly, basal autophagy was similar in the striatum and cortex in a recent study of BACHD mice (Baldo et al., 2013), suggesting that further investigations are needed to compare autophagy induction in different neuronal populations.

## SYNAPTIC ACTIVITY REGULATES PROTEOSTASIS

Cell non-autonomous pathways, such as neuronal signaling and synaptic activity, may also affect striatal proteostasis. Neuronal activity can affect levels of ubiquitinated proteins in the post-synaptic density (Ehlers, 2003) and the subcellular localization and biochemical composition of proteasomes (Bingol and Schuman, 2006; Tai et al., 2010). Neuronal stimulation can also induce autophagy (Shehata et al., 2012; Otabe et al., 2014).

The striatum receives significant dopaminergic and excitatory glutamatergic inputs from the substantia nigra and cerebral cortex, respectively. Excitotoxicity caused by glutamatergic signaling via *N*-methyl-D-aspartate receptors (NMDARs) may contribute to striatal-selective degeneration in HD (Levine et al., 1999; Zeron et al., 2002). This increased sensitivity to NMDAR activation may also affect striatal proteostasis mechanisms. Okamoto et al. (2009) showed that NMDAR extrasynaptic activity is necessary for mHtt IB formation. Dopaminergic input to the striatum, which potentiates glutamate excitotoxicity (Cepeda et al., 1998; Tang et al., 2007), also affects proteostasis. Dopamine can increase IB formation in primary neuron cultures and cell lines (Charvin et al., 2005; Robinson et al., 2008), suggesting that projections from the cortex and substantia nigra to the striatum may promote striatal susceptibility in HD by altering striatal proteostasis mechanisms (**Figure 1**).

In addition to their role as glutamate receptors, NMDARs also regulate calcium influx. Calcium dyshomeostasis can induce excitotoxicity and may cause cell death in HD models (Bezprozvanny and Hayden, 2004; Tang et al., 2005). Striatal mitochondria were found to have reduced calcium buffering capacity, and expression of calcium binding proteins in HD mouse striatal neurons was reduced, suggesting that calcium dyshomeostasis is involved in striatal-selective degeneration (Thomas, 2006; Oliveira and Gonçalves, 2009). Studies also indicate that calcium signaling can affect proteostasis. Calcium and Ca^2+^/calmodulin-dependent protein kinase II (CaMKII) can regulate UPS function and autophagy in neurons (Djakovic et al., 2009; Bingol et al., 2010; Decuypere et al., 2011). Thus, differences in striatal calcium handling may alter proteostasis capacity and induction. Overall, understanding how NMDAR, dopamine, and calcium signaling affect proteostasis will divulge cell non-autonomous mechanisms that may explain the regional selectivity of IB formation in HD.

## THERAPEUTIC IMPLICATIONS

Targeting cellular proteostasis pathways may be therapeutically beneficial in HD. **Table 1** contains a summary of proteostasis targets tested in HD models.

**Table 1:** Proteostasis targets tested in HD models.

Target	Effect	HD models tested	Phenotype improved	Reference
mTOR inhibition	Autophagy induction	Cell line, fly, mouse	Cellular toxicity, mHtt aggregation, motor phenotypes, weight gain	Ravikumar et al. (2004), Berger et al. (2006)
IMPase inhibition	Autophagy induction	Cell line	Cellular toxicity, mHtt levels	Sarkar et al. (2005)
Calpain inhibition	Autophagy induction	Cell line, zebrafish	Cellular toxicity, mHtt aggregation, photoreceptor degeneration	Williams et al. (2008)
I1R activation	Autophagy induction	Cell line, zebrafish, mouse	mHtt aggregation, photoreceptor degeneration, mHtt levels, motor phenotypes	Williams et al. (2008), Rose et al. (2010)
L-type Ca^2+^ channel inhibition	Autophagy induction	Zebrafish	mHtt aggregation, photoreceptor degeneration	Williams et al. (2008)
AMPK activation	Autophagy induction	Mouse	Motor phenotypes, survival time	Ma et al. (2007)
Hsp40 overexpression	Chaperone induction	Yeast, *in vitro*	mHtt aggregation, mHtt fibril and oligomer formation	Krobitsch and Lindquist (2000), Muchowski et al. (2000), Wacker et al. (2004)
Hsp70 overexpression	Chaperone induction	Yeast, *in vitro,* fly	mHtt aggregation, mHtt fibril and oligomer formation, ocular degeneration	Warrick et al. (1999), Krobitsch and Lindquist (2000), Muchowski et al. (2000), Wacker et al. (2004)
Hsp104 overexpression	Chaperone induction	Cell line, yeast, mouse	Cell death, mHtt aggregation, mouse survival	Carmichael et al. (2000), Krobitsch and Lindquist (2000), Vacher et al. (2005)
Hsp90 inhibition	HSF1 and HSR activation	Cell line, fly	mHtt aggregation, photoreceptor degeneration	Sittler et al. (2001), Fujikake et al. (2008)
HSF1 activation (Hsp90-independent)	HSR activation	Cell line, fly	mHtt aggregation and levels, cell death, eye degeneration	Neef et al. (2010)
Parkin overexpression	UPS induction	Cell line	PolyQ aggregation and levels	Tsai et al. (2003)
HRD1 overexpression	UPS induction	Cell line	mHtt levels and aggregation, cell death	Yang et al. (2007)
PA28γ overexpression	UPS induction	Primary neuron	mHtt levels, cell death	Seo et al. (2007)
CHIP overexpression	UPS induction	Cell line	mHtt aggregation, cell death	Jana et al. (2005)

Activating the UPS pathway is an intriguing therapeutic strategy. Overexpressing specific E3 ubiquitin ligase enzymes, such as Parkin and HrdI, increased clearance of mHtt by the UPS (Tsai et al., 2003; Yang et al., 2007). Overexpressing CHIP, a co-chaperone and a ubiquitin ligase, also reduced mHtt aggregation and cell death *in vitro* (Jana et al., 2005). Alternatively, UPS function can be induced by endogenously activating the 20S proteasome via PA700, PA200, or PA28 proteasome activators (Huang and Figueiredo-Pereira, 2010). Indeed, activating PA28γ improved cell viability in striatal neurons expressing mHtt (Seo et al., 2007) but did not improve motor phenotypes or pathology in the R6/2 mouse model (Bett et al., 2006). These results indicate that differences between *in vitro* and *in vivo* models of HD must be considered before developing effective UPS-targeting therapies.

Manipulating chaperone function may also be therapeutically effective. For example, Hsp40 and Hsp70 can reduce mHtt-dependent aggregation and toxicity (Warrick et al., 1999; Krobitsch and Lindquist, 2000; Muchowski et al., 2000; Wacker et al., 2004), while Hsp104 can reduce mHtt-induced aggregation and cell death (Carmichael et al., 2000; Krobitsch and Lindquist, 2000; Vacher et al., 2005). Furthermore, activating heat shock factor 1 (HSF1) activity, which regulates HSP expression, can suppress mHtt levels and IB formation, reduce *Drosophila* photoreceptor degeneration, and prolong lifespan of R6/2 mice (Sittler et al., 2001; Fujimoto et al., 2005; Fujikake et al., 2008; Neef et al., 2010, 2011). Chemical chaperones were also shown to be neuroprotective in HD mouse models (Tanaka et al., 2004; Gardian et al., 2005). A Phase 2 clinical trial of one such chaperone, phenylbutyrate, was completed in 2007 and demonstrated that phenylbutyrate was well tolerated in HD patients (Hersch, 2008). In 2014, the metal “chaperone” PBT2, which promoted degradation of extracellular β-amyloid by transporting metal ions into cells (Crouch et al., 2011) was examined in a Phase 2 clinical trial for HD where it was also shown to be well tolerated and had a minor positive effect on cognition (Prana Biotechnology). Further investigations must determine if results obtained in chaperone overexpression-based systems are translatable to more physiological HD models.

Finally, upregulating autophagy can ameliorate symptoms and pathology in many HD models. Inducing mammalian target of rapamycin (mTOR)-dependent autophagy reduced neurodegeneration in a fly HD model and improved behavior and motor performance in mouse HD models (Ravikumar et al., 2004; Berger et al., 2006; Sarkar et al., 2009). Inducing autophagy independently of mTOR also reduced mHtt aggregation and toxicity in various models (Sarkar et al., 2005; Ma et al., 2007; Zhang et al., 2007; Williams et al., 2008; Rose et al., 2010; Tsvetkov et al., 2010). The compounds identified in these studies act via inhibition of calpain or inositol monophosphatase (IMPase), activation of the imidazoline type 1 receptor (I1R) or AMP-activated protein kinase (AMPK), and antagonism of L-type Ca^2+^ channels. While autophagy is a promising therapeutic target, the degree of autophagy induction must be optimized if overactive autophagy is detrimental, as seen in some circumstances (Chakrabarti et al., 2009).

Over the past decade, most therapies tested in HD clinical trials have either targeted dopamine or NMDA signaling (Bonelli and Hofmann, 2007). As discussed above, both dopaminergic and NMDA signaling can affect striatal proteostasis; however, it is unclear whether the few compounds that have some effect on HD do so via proteostasis pathways. As most of these compounds do not markedly influence HD progression, it is likely that direct targeting of proteostasis pathways will be necessary to achieve clinical success.

Protein homeostasis has an important role in striatal-selective neurodegeneration in HD, and it is a strategic focus of therapeutic efforts. Since obvious symptoms of HD do not often develop until the fourth or fifth decade of life (Kieburtz et al., 1994), proteostasis pathways likely manage misfolded mHtt fairly well for a long time. Thus, future studies may find that only modestly increasing proteostasis function can stall disease indefinitely.

## CONCLUDING REMARKS

The gene responsible for HD was identified in 1993. Since then, the characteristic pathology of HD has been puzzling. If mHtt expression is ubiquitous, why do MSNs degenerate first? Although many hypotheses have emerged, the regional selectivity of IB formation indicates that differences in striatal proteostasis capacity are responsible for the selective degeneration of MSNs. Recent evidence supports this claim, demonstrating that components of autophagy, the UPS, and chaperone systems are expressed or regulated differently in striatal neurons than in other brain regions. Thus, targeting proteostasis pathways specifically in the striatum may uncover new treatments for HD.

## Conflict of Interest Statement

The authors declare that the research was conducted in the absence of any commercial or financial relationships that could be construed as a potential conflict of interest.
